# Report of Deaths in the City of Buffalo for the Month of July, 1801

**Published:** 1861-09

**Authors:** J. Whitaker

**Affiliations:** Health Physician


*Report of Deaths in the City of Buffalo for the month of July,* 1861.

Accident 15, Apoplexy 2, Cancer 1, Cholera Infantum 11, Cirrhosis of Liver 1 Consumption 17, Convulsions 14, Croup 3, Cyanosis 2, Debility 5, Delirium Tremens 3, Dentition 1, Diarrhœa 2, Disease of the Brain 2, Diphtheria 1, Dropsy of the Brain 7, Dropsy of Heart 1, Dysentery 2, Epilepsy 1, Fever 2, Fever Puerperal 2, Fever Remittent 1, Scarlet Fever 4, Typhoid Fever 1, Hæmorrhage 2, Inflammation of Bowels 4, Inflammation of Brain 3, Inflammation of Lungs 6, Inflammation of Womb 1, Intemperance 3, Disease of Kidneys 2, Marasmus 2, Measles 1, Old Age 3, Poisoning 1, Premature Birth 1, Rheumatism 1, Small Pox 3, Still Born 7, Ulcer 1, Whooping Cough 4, Unknown 10. Under one year of age, 63. Between one year and twenty, 28. Between twenty and fifty, 46. Over fifty, 19. Ages unknown, 3. Males, 97. Females, 53. Not given, 6. Total, 156.

J. WHITAKER, Health Physician.

